# The Revolving Door Phenomenon Revisited: Time to Readmission in 17’415 Patients with 37’697 Hospitalisations at a German Psychiatric Hospital

**DOI:** 10.1371/journal.pone.0075612

**Published:** 2013-10-08

**Authors:** Ulrich Frick, Hannah Frick, Berthold Langguth, Michael Landgrebe, Bettina Hübner-Liebermann, Göran Hajak

**Affiliations:** 1 Department of Psychiatry and Psychotherapy, University of Regensburg, Regensburg, Germany; 2 Döpfer University of Applied Sciences, Department of Psychology, Cologne, Germany; 3 Research Institute on Public Health and Addiction, University of Zurich, Zurich, Switzerland; 4 Department of Statistics, Universität Innsbruck, Innsbruck, Austria; 5 Department of Psychiatry, Psychosomatics, and Psychotherapy, Sozialstiftung Bamberg, Bamberg, Germany; The Nathan Kline Institute, United States of America

## Abstract

**Objective:**

Despite the recurring nature of the disease process in many psychiatric patients, individual careers and time to readmission rarely have been analysed by statistical models that incorporate sequence and velocity of recurrent hospitalisations. This study aims at comparing four statistical models specifically designed for recurrent event history analysis and evaluating the potential impact of predictor variables from different sources (patient, treatment process, social environment).

**Method:**

The so called Andersen-Gil counting process model, two variants of the conditional models of Prentice, Williams, and Peterson (gap time model, conditional probability model), and the so called frailty model were applied to a dataset of 17’415 patients observed during a 12 years period starting from 1996 and leading to 37’697 psychiatric hospitalisations. Potential prognostic factors stem from a standardized patient documentation form.

**Results:**

Estimated regression coefficients over different models were highly similar, but the frailty model best represented the sequentiality of individual treatment careers and differing velocities of disease progression. It also avoided otherwise likely misinterpretations of the impact of gender, partnership, historical time and length of stay. A widespread notion of psychiatric diseases as inevitably chronic and worsening could be rejected. Time in community was found to increase over historical time for all patients. Most important protective factors beyond diagnosis were employment, partnership, and sheltered living situation. Risky conditions were urban living and a concurrent substance use disorder.

**Conclusion:**

Prognostic factors for course of diseases should be determined only by statistical models capable of adequately incorporating the recurrent nature of psychiatric illnesses.

## Introduction

Serious mental illness is often believed to follow a natural course with chronic, recurrent episodes of the underlying disease. After the introduction of modern drug treatment (neuroleptics, antipsychotics, antidepressants) in the second half of the 20th century a formerly permanent seclusion of psychiatric patients within closed hospitals has been replaced by recurrent hospitalisations. Such recurrent hospitalisations of chronically ill patients were spread under the term “revolving door phenomenon”. In Ontario, Canada, the proportion of readmitted patients among the total of annual hospitalisations had risen from 7% in 1941 to over 50% in 1971 [1], but with stable incidence during the same period. The pessimistic model of a worsening of the course of illness with repeated hospitalisations that is associated with the term “revolving door patients” has been doubted from a population-based perspective already at that time from authors in Canada [2] [3] and later also from New Zealand [4]. The majority of patients ever hospitalised with mental health problems do not return to hospital for at least a rather long time [5] [6] [7].

But even if these “heavy users”, who display long treatment careers and multiple hospitalisations, represent only a small group from a population based perspective, they represent a considerable number among hospitalised patients and are costly. Therefore analyses on the reasons for rehospitalisation have been performed in numerous studies [8] [9] [10] [11] [12] [13]. Accurate identification of risk factors for rehospitalisation is highly relevant for several reasons. First, the anticipation of risk factors for rehospitalisation is clinically relevant when planning a patient’s discharge. Second, knowledge about course of chronic psychiatric diseases and effects of treatments is relevant for the organisation of health care systems and the allocation of public health resources. Particularly, the allocation of public funds between, e.g., in-patient and out-patient facilities requires knowledge of the trajectories of people with psychiatric disorders. Third, very recently the relevance of epidemiologic data for identifying neural mechanisms of psychiatric diseases has been stressed [14].

The majority of studies that have analysed chronification and rehospitalisation so far used either logistic regression on readmission within a certain period of time, or survival analysis for one episode only (usually the duration of the time to rehospitalisation after first discharge) as statistical models to analyse potential impact factors. However, these methods do not incorporate all information available on the disease process. Analysing the course of affective disorders, Baethge and Schlattmann [15] have demonstrated that omitting the recursive nature of the admission-discharge-process can lead to false conclusions about risk factors for readmission.

If, as a first approach to this problem, the indenture number of a single hospitalisation is taken into account, time to readmission seems to fall shorter with higher numbers of re-hospitalisations in patients with schizophrenic disorders [16], as well as with affective disorders [10]. This has been interpreted as “acceleration of the revolving door” throughout an individual treatment career. However, such an approach based on treatment populations (e.g., all schizophrenic patients after their third hospitalisation compared to all patients after their 10^th^ hospitalisation) might be misleading, when the differences of both samples are neglected. Indeed, a seemingly progressive course of schizophrenia can be explained as a selection artifact, if patients’ individual “frailties” for readmission are taken into account and integrated into the statistical analysis [17] [18]. For patients with affective disorders, both individual frailties *and* an acceleration effect over course of illness have been reported as influential [19] [10]. These individual frailties may reflect demographic characteristics, individual illness severity as well as individual variations of the treatment process and their social situation [10] [13] [12] [20]. Therefore, the study of readmission risk must take into account the individual susceptibility towards readmission.

This paper aims at analysing impact factors on the process of rehospitalisation after discharge from inpatient psychiatric treatment taking into account individual careers of patients. In detail the influence of demographic aspects, characteristics of the treatment process and socio-structural aspects have been considered.

Different statistical approaches can be used for this purpose: the counting process model [21], two slightly differing variants of the conditional probability model [22] and the frailty model (a detailed description of the different models is given in the methods section). As different statistical models for recurrent event data focus on different aspects of the event history processes, it was decided to analyse the same data set with the different models and to compare their results and conclusions that could be drawn from these analyses.

### Sample and Methods

The Psychiatric District Hospital of Regensburg serves a population of nearly 800’000 people as exclusive, single provider of inpatient psychiatric treatment in its region. Of the 27’973 patients treated at least once during the period from January, 1996 to December, 2007, all patients ever diagnosed with a F2 (schizophrenia, schizotypal and delusional disorders), F3 (affective disorders), F4 (neurotic, stress-related and somatoform disorders), F5 (behavioural syndromes with physiological disturbances), or F6 (personality disorders) ICD10 diagnosis (main diagnosis or secondary diagnosis) were selected for this study. Patients with isolated substance abuse disorders (F1) without additional diagnosis of another mental health disorder were excluded as well as patients with neurodegenerative diseases (F0) only. Co-morbidity of these disorders in this paper reflects a change in the main diagnosis over treatment episodes. N = 18’393 patients (43’891 hospitalisations) in total met this inclusion criterion. After excluding patients with either lacking admission and/or discharge dates or displaying implausible data like overlapping treatment episodes, 17’988 patients remained eligible for this study. Complete records for all covariates under study could be found in 17’145 patients, who had been treated during 37’697 hospitalisations. The latter formed the statistical basis for this study.

Patients were documented with a standardized German documentation system for psychiatric in-patient treatment (DGPPN-BADO, [23]), requiring no additional informed consent beyond the routine treatment contract. Data were anonymised and then analysed. The german law does not impose any legal restrictions on the use of anonymised data for research purpose and does not require formal approval of an ethics committee nor informed patient consent (BayDSB, http://www.datenschutz-bayern.de/verwaltung/epidem.htm, 3.3). We have received a formal waiver from the Independent Ethics Committee at the Regensburg University.

Time to readmission (TIC = time in community) was calculated as the difference between a discharge date and a subsequent readmission (or December 31, 2007, if the duration of the last TIC episode was censored). Patients displayed a mean number of 2.31 psychiatric hospitalisations (median = 1.0; maximum = 89). Median TIC duration over all episodes was 782 days (95% C.I. 742–824).

Patients’ characteristics analysed for impact on time to readmission were: patient’s sex (female episodes 49.1%), higher educational level (at least secondary level diploma = “Abitur”: 10.8% of episodes) and early onset (first psychiatric hospitalisation before 21^st^ birthday: 31.0%). These variables were registered with stable values over multiple records per person. The number of previous psychiatric hospitalisations was increased by value one for each new TIC-episode, thus reflecting the progression of a patient’s illness career. Main diagnosis per hospitalisation (dummy variables for ICD10 F-groups) and current age at discharge (median: 40 y) could vary within patients as well as status of living in a stable partnership (married and/or living with partner: 25.5% of episodes). In order to model a potential acceleration of re-hospitalisations during the course of depression reported by previous studies [10] [15], a dummy variable for this interaction effect was constructed. Social integration was additionally measured via employment status (after each discharge; at least part time employment: 13.3% of episodes) and living arrangement (“no private housing”: 14.7% of episodes). A more socio-structural aspect of the living situation was registered by classifying the place of residence as “urban” or not (cities >60.000 inhabitants: 40.6% of episodes). Further details of our study sample can be found in supporting tables S1 and S2.

Characteristics of the treatment process itself were also explored for potential impact on time to readmission: Involuntary admission to the hospitalisation preceding the current TIC-episode (16.3%), length of the preceding hospital stay (mean days = 32.6; SD 40.6), and its results (Global Assessment of Functioning = GAF score at discharge; mean = 57.7; SD 15.1) were documented for each TIC-episode. Referral to a general practitioner (39.0%) or to the hospital’s own outpatient clinic (12.0%) after discharge were chosen as variables measuring aspects of aftercare. Finally, potential historical changes in the treatment system itself, which could impact on the time to readmission, were integrated into the model by counting the historical year. The number of annual patients per year increased from 2’298 in 1996 to 3’478 in 2007 thus reflecting an ongoing trend to shorter and more frequent hospitalisations that has also been described for psychiatric inpatient treatment elsewhere [24] [7] [25].

Statistical analysis of the recurrent event process was performed using the following models:

### Counting Process Model

Andersen and Gill [21] formulated a counting process model with one process per person counting all episodes by that person. The intensity, which drives the events, is defined as
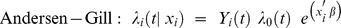
with *Y_i_(t)* indicating whether or not the person *i* with covariate vector *x_i_* is under observation at time *t*. The apostrophe denotes transposition. Baseline hazard λ_0_ and covariate effects *β* are valid for the whole sample. Due to this formulation of the intensity, Cox’s partial log-likelihood can be used to estimate the coefficients.

When treating all episodes as independent observations by different people, all episodes are moved on the time scale to start at *t = *0. The central idea of the Andersen-Gill model is that only the first episode of each person starts at *t* = 0. The subsequent episodes are kept in place (relative to their first episode). While this has no effect on the length of the time interval ( = the length of the TIC), it is reflected in *Y_i_(t)* and thus the risk sets constructed by them. Each person stays under observation until the end of their last episode– as opposed to the end of their longest episode. This ensures that the recurrence of the episodes is not ignored, but incorporated into the model.

All episodes by one person are treated as independent. Therefore, correlations between episodes by the same patient have to be accounted for by employing the robust estimate of the covariance matrix by Lin and Wei [26]. The other consequence of this independence assumption is ignoring the ordering of the recurrent events. The risk set at time *t* might include patients being at risk for, e.g., a first, third, or tenth rehospitalisation.

### Conditional Model

To avoid this mixing of risk sets, a conditional model has been proposed by Prentice and colleagues [22]. The model is conditional in the sense that patients cannot be at risk for a *j*-th event if they have not yet experienced (*j*−1) events. This condition is worked into the model by using the event number *s* as a stratum variable *s*. Within each stratum the hazard rate is modelled through a Cox model. This has been named “Prentice-Williams-Peterson Conditional Probability” (PWP CP) model. The time scale used for this model measures time continuously within each individual from day zero to the last day of observation.
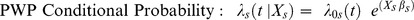



If the “gap time” version of the conditional model is used, the clock is being „reset“ to zero after each event. Within each stratum, time starts at zero for each observation belonging in this event number stratum. Thus, we are focussing on the time to event rather than on the full course of disease rsp. on the sequence of repetitive events. The model is abbreviated “PWP GT”.
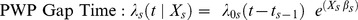



We used both slightly different versions of the PWP model. Parameter estimation is obtained by using the stratified partial likelihood. The counting process model (Andersen-Gill) and the two conditional probability models were estimated using SAS (rel.9.1) PROC PHREG.

### Frailty Model

To arrive at the so called “frailty model”, the traditional proportional hazard model of Cox is extended in its parametric part to model recurrent events of the same person *i*. The correlation between *n_i_* episodes of the same patient *i* is interpreted as an individual characteristic of that person and expressed through an additional term γ_i_ to be included into the Cox model,
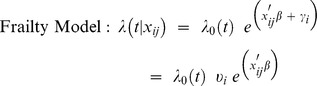
where *i = 1, …, n* represents the number of the person and *j = 1, …, n_i_* represents the number of the episode of person *i*.

It is common to assume the frailty parameters to be Gamma distributed with the scale and shape parameter estimated from the data. All episodes by one person share the same frailty term, which incorporates the correlation between these episodes into the model.

While the effects of the covariates are valid for all patients, the frailty term modifies the baseline hazard to individual levels. Patients with a high frailty have a high risk of recurrence during all of their episodes. The model assumes “baseline velocities” of the disease process to vary between patients. Estimation for this study was done via the approach of Therneau and colleagues [27] who formulate the Cox model with a shared gamma frailty as a penalized model to reduce computational complexity. Calculations were performed in R [28] using the package “survival” [29].

Beyond these four models, in a further model only the first TIC episode of each patient was analysed by a traditional proportional hazard model as an alternative to control for the influence of the course of the disease. As the indenture number of the TIC episode does not vary in this subset, a potential interaction between diagnosis F3 and course of disease could not be estimated.

## Results

**Figure 1:**
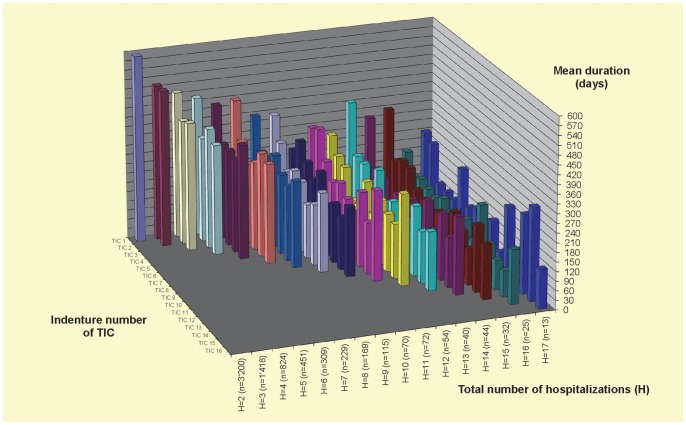
Duration of “Time in Community” (uncensored episodes 1998–2007) by total number of hospitalisations and indenture number of TIC episode.

Figure 1 gives a descriptive overview on mean durations of completed episodes of “time in community” by indenture number of TIC and total number of hospitalisations observed. With respect to the indenture number of TIC episodes, a prominent reduction of mean durations can be observed between the first (mean = 508.6 days; SD = 690) and all subsequent episodes (mean = 249.8; SD = 419), no matter how many hospitalisations were observed in total. Between the second and all later TIC episodes (from the rear to the front bars in figure 1) there is no clear trend of shortening or prolongation of durations, though there is considerable variation.

On the other hand, a trend towards shorter initial TIC durations can be observed with increasing total number of hospitalisations (last row of bars in figure 1 from the left to the right). This points to inter-individual differences of the underlying disease process. There exists a group of patients with an accelerated disease process, who clearly have shorter TIC durations, also after their initial hospitalisation, and therefore are at higher risk to collect more hospitalisations during a given observation period. Results for patient groups with higher numbers of hospitalisations (> = 9) are based on rather small samples and therefore are subject to higher impact of random variation.

Durations of recurrent TIC episodes (including censored episodes) were regressed by the different models listed in Table 1. Analyses yielded consistent results: The estimated coefficients for the various predictor variables were highly similar and reached a correlation of at least 0.90 between all pairs of models. However, we also observed relevant differences in the results depending on the model used. This was the case for the patient related factors “sex” and “living in partnership”, and also for the factor “historical time”. The relevance of length of hospitalisation depended on whether only the first episode was considered (Cox-regression-model) or whether all episodes were analysed (all other models).

**Table 1:** Regression coefficients of the five statistical models estimated to predict recurrent rehospitalisations.

Predictor Variable	Risk Ratio (95% C.I.)				
	Counting Process Model Andersen-Gill	ConditionalModelPWP-CP	ConditionalModelPWP-GT	Frailty Model	Cox Regression 1^st^ episode only
Sex = female	0.987 (0.929–1.048)	1.007 (0.967–1.048)	1.035 (1.001–1.069)	1.037 (1.002–1.082)	1.015 (0.964–1.068)
Current diagnosis = F1	1.513 (1.409–1.623)	1.202 (1.141–1.266)	1.296 (1.246–1.349)	1.384 (1.318–1.452)	1.448 (1.351–1.552)
Current diagnosis = F3	0.597 (0.549–0.649)	0.743 (0.698–0.792)	0.866 (0.822–0.912)	0.824 (0.776–0.876)	0.865 (0.806–0.929)
Interaktion F3 with Course of Illness (# hosp.)	1.046 (1.021–1.072)	1.046 (1.030–1.063)	1.014 (1.007–1.022)	1.016 (1.008–1.025)	n.a.
Current diagnosis = F4	0.398 (0.367–0.431)	0.556 (0.519–0.596)	0.612 (0.574–0.652)	0.569 (0.533–0.607)	0.554 (0.508–0.604)
Current diagnosis = F5	0.381 (0.235–0.616)	0.543 (0.373–0.790)	0.586 (0.396–0.868)	0.533 (0.397–0.715)	0.537 (0.375–0.771)
Place of residence = urban	1.436 (1.342–1.536)	1.192 (1.143–1.243)	1.159 (1.121–1.197)	1.254 (1.207–1.307)	1.103 (1.046–1.163)
Spouse/partner	1.000 (1.000–1.000)	1.000 (1.000–1.000)	1.000 (1.000–1.000)	0.915 (0.874–0.958)	1.000 (1.000–1.000)
Compulsory hospitalisation	0.839 (0.785–0.898)	0.946 (0.901–0.993)	0.920 (0.886–0.956)	0.900 (0.860–0.941)	0.997 (0.916–1.043)
Higher education	0.812 (0.739–0.839)	0.909 (0.854–0.968)	0.924 (0.876–0.976)	0.871 (0.821–0.925)	0.896 (0.827–0.971)
Age at discharge	0.996 (0.994–0.998)	0.996 (0.995–0.998)	0.992 (0.991–0.993)	0.992 (0.991–0.994)	0.996 (0.994–0.998)
Employment after discharge	0.683 (0.636–0.732)	0.832 (0.784–0.884)	0.889 (0.843–0.936)	0.849 (0.803–0.899)	0.830 (0.765–0.901)
GAF score at discharge	0.986 (0.984–0.988)	0.993 (0.991–0.994)	0.992 (0.991–0.993)	0.990 (0.988–0.991)	0.989 (0.988–0.991)
No private housing after discharge	0.759 (0.641–0.898)	0.725 (0.635–0.829)	0.887 (0.806–0.976)	0.875 (0.797–0.961)	0.810 (0.682–0.961)
Referral to hospital’s outpatient clinic	1.507 (1.386–1.638)	1.188 (1.117–1.263)	1.114 (1.066–1.163)	1.143 (1.087–1.201)	1.072 (0.980–1.173)
Referral to general practitioner	0.936 (0.888–0.987)	0.962 (0.923–1.003)	0.960 (0.931–0.991)	0.945 (0.913–0.977)	0.989 (0.983–1.043)
Length of stay (before last discharge)	0.999 (0.998–0.999)	0.999 (0.999–1.000)	0.999 (0.999–1.000)	0.999 (0.999–0.999)	1.002 (1.001–1.002)
Historical year (discharge)	1.055 (1.046–1.065)	1.009 (1.003–1.016)	0.949 (0.943–0.954)	0.970 (0.964–0.976)	0.994 (0.986–1.003)
Indenture number of hospitalisation	1.081 (1.071–1.091)	stratum	stratum	1.004 (1.002–1.007)	1.080 (1.078–1.083)§

§ For this model, the predictor variable used is the total number of psychiatric hospitalisations.

A (transient or incident) co-diagnosis of any substance abuse disorder (F1) was consistently associated with an accelerating effect (odds ratios between 1.20 and 1.51) on rehospitalisation throughout all models. Affective disorders (F3) were connected with longer TIC episodes (OR between 0.60 and 0.87), no matter which model is estimated. But the longer the course of illness, this “protective” effect of affective disorders is more and more counter-balanced by a significant interaction between diagnosis and number of hospitalisations, thus replicating the results of [19] [10]. Neurotic and somatoform disorders (F4) as well as behavioural syndromes (F5) displayed longer TIC episodes and therefore less frequent readmissions as compared to the reference group (F2 or F6 ∶29.1%+9.7% of episodes). Effect sizes were quite large and estimated at odds ratios between 0.38 and 0.61. Another characteristic of the disease process, “first psychiatric hospitalisation before age of 21”, did not reach statistical significance as a predictor for time in community under any of the models and therefore was not included in table 1.

Patients’ socio-demographic characteristics were not always found to have homogeneous effects over statistical models. Age at discharge and higher education throughout all models were estimated as protective variables associated with longer TIC episodes. A potential accelerating effect of female sex on readmission could only be shown in the PWP gap time and the frailty model, and a protective effect of being married (rsp. living in partnership) was only significant in the frailty model.

Aspects of a patient’s social situation after discharge were found to be influential in all models: Living in an urban surrounding was associated with a higher risk of rehospitalisation (OR from 1.16 to 1.44), and living in an institutionalized or precarious setting (“no private housing”) with a diminished risk for rehospitalisation (OR between 0.76 and 0.89). Patients’ social functioning at the time of discharge (as measured by the GAF score) displayed a protective effect for re-hospitalisation in all models, as well as employment.

Involuntary hospitalisation could be shown to be associated with delayed and less frequent rehospitalisation (OR between 0.84 and 0.95). Referral to a general practitioner was associated with longer TIC episodes, and referral to the hospital’s own outpatient clinic with significantly shorter TIC episodes. Longer lengths of stay were homogeneously found to be significantly connected with longer subsequent TIC episodes over all statistical models except the 1^st^-episode-only-Cox-regression-model. In all models, where the number of lifetime psychiatric hospitalisations could be integrated (not possible for the two conditional PWP models), a positive association between number of hospitalisations and accelerated rehospitalisation could be found. Finally, a rather contradictory effect was estimated for the historical time: Whereas the Andersen-Gil counting process model and the PWP conditional probability model estimated a significant increase in rehospitalisation risk and shorter TIC episodes with progression of historical time, the PWP gap time model, the frailty model, and the 1^st^-episode-only-Cox-regression model came to the contrary conclusion. With ongoing historical time, these models found a decrease in rehospitalisation risk and a prolongation of TIC episodes.

## Discussion

The results of this study should be discussed from two different perspectives: From a methodological viewpoint the question should be answered, which model(s) seem(s) best suited for the specific situation of analysing recurrent hospitalisations in psychiatry. From a substantive viewpoint, subsequently the essential conclusions might be drawn about prognostic factors for rehospitalisation.

Recurrent hospitalisations in a psychiatric hospital represent a sequence of two states (hospitalised/in community) of which the indenture number of the respective hospitalisation indicates disease progression and all episodes of the same person are correlated. Biographical age of a person is for all episodes included as a prognostic factor that progresses over episodes. Period effects are represented by historical time in all models. An effect of early onset (first hospitalisation before age 21) was not present in our data.

### Methodological Aspects

The counting process model addresses the sequence aspect of recurrence by keeping the episodes in one timeline. However, it does not incorporate the correlation of episodes by one patient but rather corrects for it. As mentioned in the methods section, this model formulation results in a mixing of risk sets which is avoided by the conditional models. The conditional models both divide the episodes into strata, based on their indenture number, which are used to estimate specific parameters for prognostic factors in different disease stages. The precision of those estimates depends on the number of observations in the corresponding stratum. Precision thus can be rather low, especially for strata with higher indenture numbers. The frailty model deals with the intra-person correlation aspect of recurrence by including a frailty term directly in the model (rather than correcting for it afterwards). The sequentiality aspect of recurrence is indirectly included by adding the indenture number as a covariate. Prognostic factors are modelled as independent of disease progression (in contrast to the conditional models), but might be tested for interaction with disease progression by including the respective terms (analogously to the interaction of depression with number of hospitalisation in table 1). The frailty parameter can be interpreted as the individual velocity of the disease progression. However, no types of disease trajectories (e.g. chronic-relapsing vs. chronic-progressive) are modeled. This would require a mixture distribution assumption for the frailties (e.g. [30]). An overview on rationale and interpretation of the models is given in table 2.

**Table 2:** Characteristics and (Dis)Advantages of the Statistical Models under Comparison.

	Representation of …		Interpretational …	
Model	Sequentiality	Intra-person correlation	Disadvantages	Merits
**Counting Process Model** **Andersen-Gill**	one timeline for allepisodes	not representated, but parameter estimates corrected	mixing of risk sets	extends Cox-model to incorporate sequentiality
**Conditional Model PWP-CP (conditional probability)**	strata based on indenturenumber; continuous timeline	conditioned out via stratification	loss of precision for small strata (e.g. higher indenture numbers)	avoids mixing of risk sets
**Conditional Model PWP-GT** **(gap time)**	strata based on indenturenumber; clock reset to zero	conditioned out via stratification	loss of precision for small strata (e.g. higher indenture numbers)	avoids mixing of risk sets
**Frailty Model**	indenture numberas covariate	represented by person-specific parameter togovern base velocity ofdisease process	assumes common distributionof disease velocities; impedes identification of disjunctdisease trajectories	intuitively convincing representation of intra-person correlation
**Cox Regression only Tic1**	artificially suppressed	not representable	excludes course of illness	avoids mixing stages of disease progression

Selection of the appropriate model was postulated primarily as a question of study aims and data structure [31] rather than the application of a single fit measure (e.g. information criteria BIC, AIC etc. or likelihood ratio tests in case of nested models). We are not aware of a straightforward measure that would easily allow for comparing model fits across also stratified regression approaches. For statistical inference on predictor variables in recurrent event analysis “it is natural to approach the data using random effects” ([32], p.385), i.e. using the frailty model. We argue that the frailty model deals best with the correlation aspect of recurrence and includes the sequence aspect of disease progression in a satisfactory manner. By using a frailty approach, we additionally may overcome a former debate in the literature on logistic regression models of the readmission risk, which cut-off point in TIC duration should be set to characterize a readmission as indicating suboptimal discharge management (see [33] for an overview). Readmissions beyond this deadline of e.g. 21 or 30 days often have been neglected, because they allegedly measure characteristics of the community aftercare, not the quality of inpatient treatment. If the fact that patients display different velocities of their disease progression is adequately taken into account, a distinction between “early, unplanned, inadequate, etc.” and “neglectable” readmission as two different study endpoints becomes obsolete.

### Substantive Aspects: Patient Level Effects

Contradictory results emerged for the effects of sex and spouse/partner as individual prognostic factors. Adjusting for intra-person correlation increases precision of estimates (e.g. [32] for a simulation study) and thus yields significant results for sex and stable partnership in the frailty model.

Lower rehospitalisation rates for F3, F4 and F5 diagnosis as compared to F2 or F6 reflect the tendency towards a chronic course for schizophrenia and personality disorders as compared to other mental disorders [34] [35] [12]. It seems noteworthy that early onset in our sample was not related to course of illness and/or rehospitalisation risk. The impact of early onset on the prospective course of many disorders has been described in various studies (e.g. for F2: [36]; for bipolar disorder: [37], [38]; for major depression [39]). It might be speculated, whether the omission of the recursive nature of the disease process in most statistical approaches so far is responsible for this difference, and/or whether the inclusion of a relatively broad spectrum of prognostic variables into the statistical model has already partialised out the impact of age at onset on risk of recurrence. For the case of depression, a recent study could demonstrate this latter effect (age at onset not independently impacting recurrence in a multivariate approach) in a representative Dutch cohort study [40]. Based on previous literature, which suggests an increase of rehospitalisation risk over the course of affective disorders [19] [10] [20], we also determined the interaction of F3 diagnosis and disease course, where we could confirm such an “acceleration effect”.

All models revealed a highly increased risk of readmission in patients with co-morbid substance use disorders, a finding that is also in accordance with the literature [35] [12] [41]. Comparable to earlier studies [13] [8], a better psychosocial functional level at discharge, as reflected by a higher GAF score, was related to longer TIC episodes and less frequent readmissions.

In accordance with earlier studies, a beneficial effect of “employment” and “living in a partnership” has been found by the frailty model [9]; [42]; [12]; [43]. It should be noted, however, that in an earlier study [9] the effect of living alone was modulated by the employment situation and age. In light of the large number of predictor variables analysed in our study, the inclusion of all potential two-way and higher-order interactions would have made a meaningful analysis impossible. Therefore, we restricted ourselves to a priori formulated interaction effects of lower order that had been described in the literature prior to our study.

Whereas “early onset” had no independent influence on admission rate, patient’s age at discharge had an effect, with higher age being related to a lower readmission risk. Most previous studies investigating risk factors for repeated hospitalisation did not analyse the role of age or did not find an effect for age. One study suggests that the interaction of age and living situation has an effect on hospital readmission rates [9]. Evaluating patterns of mental health care utilization, one study is in line with our results that patients with one admission only were significantly older than patients with multiple admissions [13]. Similar to previous studies [8] [44], we could identify “higher education” as a protective factor preventing readmission.

### Substantive Aspects: Effects of the Treatment Process

The significant, but seemingly contradictory results for historical year might be explained by an “over-fitting” of time. The gap-time model, the frailty model and the Cox model all reset the clock for each TIC episode to start at time zero. Therefore, any changes over historical time are fully attributed to the respective variable. By contrast, the Andersen-Gil model and the conditional probability model both incorporate historical time partly in their internal time line (as it is not reset to zero). Consequently the variable “historical year” represents the deviation of a full period effect from individual disease progression, which cannot be interpreted straightforwardly.

All models analysing all episodes suggest a protective effect of length of stay. However, this is not the case if only the first episode is considered as in the Cox-model. Taken together, these considerations suggest that the length of the first hospitalisation has no relevance for the rehospitalisation risk. By contrast, in repeatedly hospitalised patients a longer length of stay predicts a longer time in community. A potential explanation for this finding could be the existence of two distinct disease patterns: acute diseases with one episode and complete recovery on one side, and chronic diseases with repeated episodes on the other side. A longer hospitalisation would only have a protective effect in the second group, and is submerged at the first hospitalisation by the “one episode only” group. Such a distinction may be implicitly reflected by healthcare routines in mental health hospitals, where a switch from an “acute care mode” to a “chronic care mode” could be identified when patients are readmitted [24]. Based on our results it is tempting to speculate that in the group of chronic but not of acute patients an “investment” in longer LOS seems worthwhile. However, this hypothesis needs confirmation by interventional studies, before firm conclusions can be drawn.

Our results could also provide an explanation for conflicting results in the literature about the impact of length of hospital stay on the course of illness. Although some authors have reported lower relapse risk and better outcome with shorter hospital stays ( [45], [46], [9]), others have found either better outcomes with longer hospital stays [47], [48] or no relationship between length of stay and course of illness [49].

Compulsory hospitalisation had a significant positive effect on the subsequent time in community. This replicates the results of an earlier study, in which psychotic patients with repeated hospitalisations had longer stays in community after compulsory admission as compared to non-compulsory admissions [50]. Whether these longer time periods in community are a consequence of more efficient treatment or rather the results of avoidance of in-patient treatment after the experience of a compulsory admission remains an open question.

Referral to the hospital’s outpatient clinic was related to an increased risk of early readmission, whereas referral to the general practitioner was related to a reduced risk. Presumably this result reflects a selection effect: Patients with a poor prognosis and a high readmission risk (according to the discharging physician’s judgment) were preferably referred to the hospital’s outpatient clinic, whereas those with a favourable prognosis were referred to the general practitioners. However, it has to be considered that this judgmental effect was significant, even if our model corrected for relevant prognostic variables such as diagnosis, GAF score and employment. Therefore it cannot be excluded that the intensive treatment in the hospitals outpatient clinic is less effective for preventing readmission than outpatient treatment by GPs. For disentangling selection and treatment effects prospective randomized trials are needed. But available evidence suggests that intense psychosocial support, as provided by the hospital’s outpatient clinic, prevents readmissions [51].

### Substantive Aspects: Effects of the Social Environment

In our study institutionalized living (as compared to private housing) had a protective effect preventing further hospitalisations. In the literature there are conflicting results with respect to the role of independent versus institutional living [12] [52]. These discrepant effects may be related to different forms of institutional housing. Therapeutic communities in a systematic review have been shown to represent an efficient long term therapeutic intervention [53].

A relevant effect on rehospitalisation risk has been found for “urban living”. Whereas a clearly increased risk for developing schizophrenia in people brought up in urban environments is well known [54], we are not aware of any studies that also identified urban living as a risk factor for higher rehospitalisation rates or a more severe course of the disease. Notably we defined “urban living” at a low threshold (60.000 inhabitants) and yet were able to detect this effect. Based on our data we cannot delineate to which extent this effect is driven by the so-called “drift hypothesis” or by increased stress related to urban living. Recent functional imaging studies have demonstrated that urban living is associated with increased amygdala activity during social evaluative stress processing in humans [55]. It is conceivable that the disturbed functionality of brain circuits, which regulate negative emotions and stress represents a risk factor not only for the development of schizophrenia but also for the course of psychiatric disorders in general.

The frailty model demonstrates a clear tendency towards longer time periods in community over the time period between 1996 and 2007. This could be due to improved out-patient service offers (e.g. local day hospitals), but might also be related to a switch in pharmacologic treatment routines in these years from typical to atypical neuroleptics.

### Strengths and Limitations

Beside the use of various statistical models, the relative large sample size and the long observation period for the analysis of recurrent events, our study has the strength that not only patients with schizophrenia or affective disorders were analysed, but also patients with neurotic, stress-related and somatoform disorders (F4) and behavioural syndromes (F5). Moreover, the data analysed come from a hospital, which is the exclusive provider of in-patient treatment for a catchment area of about 800’000 inhabitants with a low rate of population movement. Thus, no selection bias due to different provider profiles seems probable. Nevertheless, our study clearly is not free of limitations.

First, 2.2% of all patients (n = 405) displayed implausible data (e.g. overlapping inpatient treatment episodes) and therefore had been precluded from the analysis. One could speculate that such patients are mainly the consequence of erroneous homonyms with regard to their patient identification variable, rather than representing false dates for the hospitalisation period, as the latter variables are cross-checked via the accounting system of our hospital. If we assume that only false homonyms were recognized that had hit another patient’s treatment episode, and their occurrence could even be more frequent, then an overestimation of the number of re-hospitalisations and thus of velocities of the disease process might be possible.

Second, for each patient in our data set the duration of their last TIC-episode is censored. However, some patients might have moved outside the catchment area (and been treated elsewhere) or might have deliberately chosen another hospital for further inpatient treatment. This artificially prolongs the measured duration of their last TIC-episode in our data set. Especially, if the patient has deceased after their last discharge, an inflated estimate of the impact of age on TIC cannot be excluded because the probability of death increases also with age.

Third, biased estimates due to “informative censoring” [56] on missing predictor variables might be possible. Though we [57] have shown for the first years of our observation period that missing values in the standardized documentation system in our hospital were not hampering the statistical analysis of essential epidemiological results, there is no similar analysis available for the years since 2000. Consequently, a rate of 6.8% of all patients with lacking or implausible data should be kept in mind while interpreting the substantive results.

Finally, the inherent limitation of the observational design with data stemming from a single provider has to be considered while interpreting our results. For the detection of causal relationships between specific treatment factors like length of stay in hospital or specific forms of outpatient treatment prospective randomized interventional studies are needed.

To summarise, by analysing readmission patterns of mentally ill patients, our study was able to demonstrate that the choice of the statistical model is relevant and that the frailty model has advantages compared to other approaches. Logistic regression and Cox regression of one episode only are not capable of adequate capturing the nature of the disease process in psychiatric patients.

In our large sample we could confirm that there is no general acceleration effect of hospitalisations, but a certain tendency towards an acceleration in patients with affective disorders. Most identified risk factors for re-hospitalisation (diagnosis, low GAF score, no higher education, unemployment) are in accordance with the literature. A relevant new result was the identification of “urban living” as an independent potential risk factor not only for developing a specific disease (schizophrenia), but also for the course of illness of various psychiatric diagnoses. Finally, a differential role of length of stay of first versus subsequent hospitalisations for readmission risk could be reconfirmed.

## Supporting Information

Table S1 Diagnoses at Discharge (ICD10) by Indenture Number of Hospitalisations.
(XLSX)

Table S2 Sociodemographic characteristics.
(XLSX)
