# Correction: The Revolving Door Phenomenon Revisited: Time to Readmission in 17’145 Patients with 37’697 Hospitalisations at a German Psychiatric Hospital

**DOI:** 10.1371/annotation/34e8bb9b-68f4-4e96-947d-24a51f6c2665

**Published:** 2013-12-09

**Authors:** Ulrich Frick, Hannah Frick, Berthold Langguth, Michael Landgrebe, Bettina Hübner-Liebermann, Göran Hajak

The incorrect number of patients is indicated in the title of the article. 17’415 should be 17’145. Please see the corrected title here:

The Revolving Door Phenomenon Revisited: Time to Readmission in 17’145 Patients with 37’697 Hospitalisations at a German Psychiatric Hospital

This error also occurred in the first sentence of the Methods section. Please see the corrected sentence here, with 17,145:

"The so called Andersen-Gil counting process model, two variants of the conditional models of Prentice, Williams, and Peterson (gap time model, conditional probability model), and the so called frailty model were applied to a dataset of 17’145 patients observed during a 12 years period starting from 1996 and leading to 37’697 psychiatric hospitalisations." 

